# Homeotic Genes and the ABCDE Model for Floral Organ Formation in Wheat

**DOI:** 10.3390/plants2030379

**Published:** 2013-06-25

**Authors:** Koji Murai

**Affiliations:** Department of Bioscience, Fukui Prefectural University, 4-1-1 Matsuoka-kenjojima, Eiheiji-cho, Yoshida-gun, Fukui 910-1195, Japan; E-Mail: murai@fpu.ac.jp; Tel.: +81-776-61-6000 (ext. 3618); Fax: +81-776-61-6015

**Keywords:** ABCDE model, floral organ, homeotic gene, MADS-box gene, pistillody, wheat

## Abstract

Floral organ formation has been the subject of intensive study for over 20 years, particularly in the model dicot species *Arabidopsis thaliana*. These studies have led to the establishment of a general model for the development of floral organs in higher plants, the so-called ABCDE model, in which floral whorl-specific combinations of class A, B, C, D, or E genes specify floral organ identity. In *Arabidopsis*, class A, B, C, D, E genes encode MADS-box transcription factors except for the class A gene *APETALA2*. Mutation of these genes induces floral organ homeosis. In this review, I focus on the roles of these homeotic genes in bread wheat (*Triticum aestivum*), particularly with respect to the ABCDE model. Pistillody, the homeotic transformation of stamens into pistil-like structures, occurs in cytoplasmic substitution (alloplasmic) wheat lines that have the cytoplasm of the related wild species *Aegilops crassa.* This phenomenon is a valuable tool for analysis of the wheat ABCDE model. Using an alloplasmic line, the wheat ortholog of *DROOPING LEAF* (*TaDL*), a member of the *YABBY* gene family, has been shown to regulate pistil specification. Here, I describe the current understanding of the ABCDE model for floral organ formation in wheat.

## 1. Introduction

The ABCDE model for flower development proposes that floral organ identity is defined by five classes of homeotic genes, named A, B, C, D and E [1]. According to the floral quartet models of floral organ specification [2], the A- and E-class protein complex develop sepals as the ground-state floral organs in the first floral whorl, the A-, B- and E-class protein complex specify petals in the second whorl, the B-, C- and E-class protein complex specify stamens in the third whorl, and the C- and E-class protein complex specify carpels in the fourth whorl. Cloning of ABCDE homeotic genes in *Arabidopsis* showed that they encode MADS-box transcription factors except for the class A gene, *APETALA2* (*AP2*) [3]. In *Arabidopsis*, the class A MADS-box gene is *AP1* [4], the class B genes are *AP3* and *PISTILLATA* (*PI*) [5,6], the class C gene is *AGAMOUS* (*AG*) [7], and the class D genes are *SEEDSTICK* (*STK*), *SHATTERPROOF1* (*SHP1*) and *SHP2* [8,9]. The D-class proteins interact in larger complex with the E-class proteins to specify ovule identity. In the *Arabidopsis* genome, four class E genes have been found, *SEPALLATA1* (*SEP1*), *SEP2*, *SEP3* and *SEP4*, which show partially redundant functions in identity determination of sepals, petals, stamens and carpels [10,11]. The diversification of MADS-box genes during evolution has contributed to the wide variation of flower forms in angiosperms [12]. Although they are not included in the conventional ABCDE model, the *AGAMOUS LIKE 6* (*AGL6*)-clade genes *AGL6* and *AGL13*, may play a role in floral organ formation, probably in ovule formation [13]. *AGL6*-clade genes comprise a sister clade of *SEP* genes and may share an E class function with *SEP* genes.

**Figure 1:**
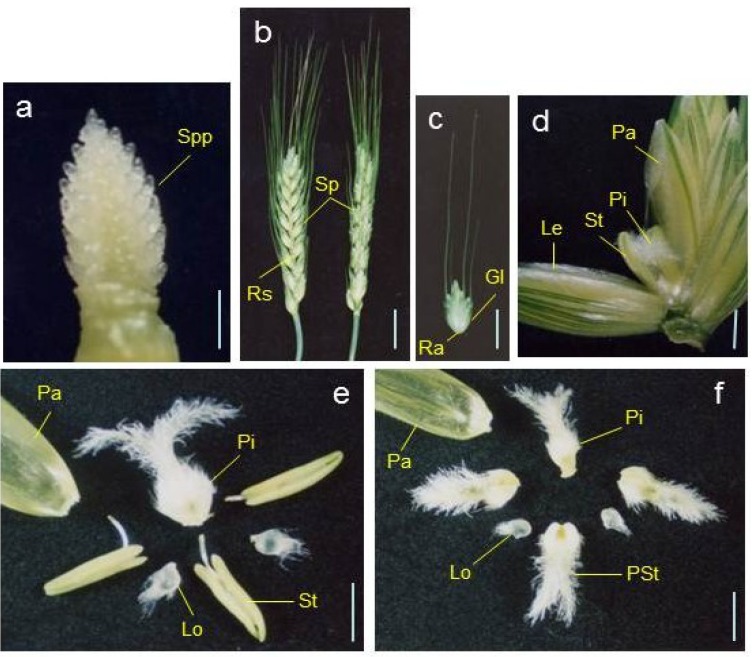
Wheat inflorescences and floral organs. (**a**) Developing young spike at the floret differentiation stage. The spikelet primordium (Spp) is indicated. Scale bar = 1 mm; (**b**) The wheat inflorescence (spike, ear, or head) is composed of spikelets (Sp) attached at the nodes of a zigzag rachis (Rs). Scale bar = 2 cm; (**c**) A spikelet that has been removed from the rachis. The spikelet consists of multiple (usually six to eight) florets attached at the rachilla (Ra). Two small bract leaves called glumes (Gl) enclose the spikelet. Scale bar = 1 cm; (**d**) A magnified image of an opened floret. In the floret, the reproductive organs, pistil (Pi) and stamens (St) are enveloped by two leaf-like structures, the lemma (Le) and the palea (Pa). The lemma and palea have been separated to make the reproductive organs visible in the figure. Scale bar = 2 mm; (**e**) An individual flower containing one pistil (Pi), three stamens (St) and two lodicules (Lo). The palea (Pa) is also indicated. In this figure, the pistil, stamens, lodicules and palea have been removed from the rachilla. Scale bar = 2 mm; (**f**) A flower from a plant of the pistillody line. The stamens are transformed into pistil-like structure (Pst) with stigmas. Scale bar = 2 mm.

**Figure 2:**
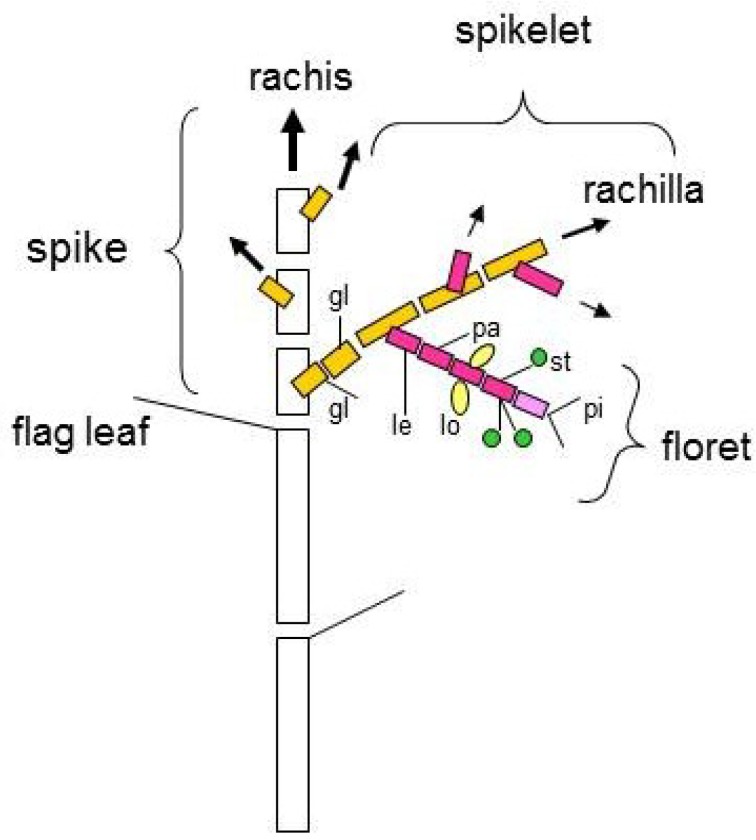
Schematic illustrations of the phytomeric structures of the wheat inflorescence. The spikelets are arranged as two opposite rows of lateral branches from the main axis (rachis). Each spikelet is composed of florets joined at the axis (rachilla) alternately on opposite sides, and enclosed by two glumes. Each floret is composed of a lemma, a palea, two lodicules, three stamens and a pistil. gl, glume; le, lemma; pa, palea; lo, lodicule; st, stamen; pi, pistil.

Grass species, such as rice (*Oryza sativa*), wheat (*Triticum aestivum*) and maize (*Zea mays*), form a unique reproductive inflorescence unit termed a spikelet [14,15]. The spikelet is comprised of florets and is encompassed by two small bract leaves (called glumes in wheat). Inflorescence development in wheat involves a series of stages: first, the inflorescence meristem produces a spikelet meristem as an axillary meristem; next, the spikelet meristem produces a floret meristem as an axillary meristem; finally, the floret meristem produces the floral organs (Figure 1, Figure 2) [16,17]. Development of the inflorescence in maize and rice is more complicated than in wheat because of the presence of additional axillary branch meristems: the tassel branch and spikelet pair meristem in maize, and the panicle branch meristem in rice [17,18]. In wheat, the spikelet is composed of florets that join the axis (rachilla) alternately on opposite sides, and is encompassed by two glumes (Figure 1, Figure 2). Each spikelet usually has six to eight florets, some of which, in apical positions, can be sterile due to hypoplasia. In each floret, the reproductive organs are enveloped by two leaf-like structures, a lemma and a palea. The lemma and palea are considered to have different origins. The lemma is a bract, which is a leaf subtending the axillary meristem of the spikelet axis; the palea is a prophyll, which is the first leaf formed by the axillary meristem [19]. An individual wheat flower contains one pistil, three stamens and two lodicules. The pistil, which is probably composed of three fused carpels, is the female part of the flower and consists of the ovary containing the ovule and two filamentous styles, each terminating with a feathery stigma. The stamen is composed of a filament and an anther containing pollen grains. Lodicules are attached to the ovary, and swell during anthesis forcing the lemma and palea apart to facilitate pollination of the stigma from the dehisced anther. There is evidence that the development of lodicules in rice and petals in *Arabidopsis* are regulated by a similar mechanism [20], suggesting that the lodicule was originally a modified petal. In summary, a palea, lodicules, stamens and a pistil are wheat floral organs developed in the whorl 1, 2, 3, and 4, respectively. Analysis of ABCDE genes in monocot species such as rice suggests that the ABCDE model might equally apply to monocots [18,21]. Here, we focus on application of the ABCDE model to flower development in wheat.

## 2. Summary of ABCDE Model in Rice

### 2.1. Rice Class A Genes

Three *AP1*-like MADS-box genes have been identified in the rice genome, namely, *OsMADS14*/*RAP1B*, *OsMADS15*/*RAP1A* and *OsMADS18*, which are all derived from the *FRUITFULL* (*FUL*) lineage rather than *AP1* as in *Arabidopsis* [22]. Studies on transgenic plants suggested that *OsMADS14* is involved in promoting flowering and in determining the identity of the floral meristem [23]. Interestingly, analysis of the *OsMADS15* mutant *degenerative palea* (*dep*) indicated that *OsMADS15* plays a role in palea formation [24]. On the basis that the palea of rice, rather than the lemma, is evolutionarily identical with the sepal of *Arabidopsis*, then *OsMADS15* is likely to be a rice class A gene. However, the *dep* mutation does not cause defects in the lodicules [24], suggesting that lodicule specification is controlled by another class A gene, an *AP2*-like gene. It has been reported that overexpression of micro RNA miR172, a negative regulator of *AP2*, results in the conversion of lodicules into the palea marginal region in transgenic rice plants [25]. Recently, two *AP2*-like genes, *SUPERNUMERARY BRACT* (*SNB*) and *Os INDETERMINATE SPIKELET1* (*OsIDS1*), were identified in rice to be required for lodicule development [26]. *SNB* and *OsIDS1* are positively regulated by another *AP2*-like gene, *MULTI-FLORET SPIKELT1* (*MFS1*) [27]. Furthermore, these rice *AP2*-like genes determine inflorescence architecture by regulating changes in spikelet meristem fate.

### 2.2. Rice Class B Genes

*OsMADS2* and *OsMADS4* have been reported to be the rice orthologs of *PI* and to have been generated by an ancient gene duplication event [28]. RNAi suppression of *OsMADS2* results in homeotic change to lodicules but stamens still develop normally [29]. By contrast, RNAi suppression of *OsMADS4* does not induce any alterations to either lodicules or stamens [30], although simultaneous loss-of-function in both *OsMADS2* and *OsMADS4* results in conversion of lodicules to palea-like organs and stamens to carpel-like organs [30]. These observations indicate that *OsMADS2* plays a more important role than *OsMADS4* in lodicule specification, and that *OsMADS2* and *OsMADS4* have an equal function in stamen formation.

*OsMADS16* /*SUPERWOMAN1* (*SPW1*) is the sole *AP3* ortholog in the rice genome [31]. A yeast two-hybrid assay indicated that *OsMADS16* interacts with both *OsMADS2* and *OsMADS4* [32]. Loss-of-function of *OsMADS16* causes the same phenotype as RNAi-mediated simultaneous suppression of *OsMADS2* and *OsMADS4*, *i.e.*, the conversion of lodicules and stamens into palea-like and carpel-like organs, respectively [31]. Overall, these findings indicate that the two *PI*-like genes, *OsMADS2* and *OsMADS4*, and the *AP3*-like gene, *OsMADS16*, are class B genes in rice.

### 2.3. Rice Class C Genes

The duplicated class C genes in rice, *OsMADS3* and *OsMADS58*, have been reported to show partial conservation of function with the *Arabidopsis* class C gene, *AG*. Mutant and transgenic analyses indicated that *OsMADS3* predominantly regulates stamen identity and prevents lodicule development and that *OsMADS58* regulates floral meristem determinacy and normal carpel morphogenesis [33]. However, a recent study on *OsMADS3* and *OsMADS58* mutants suggested that the two genes redundantly mediate the *C*-function and, together with *OsMADS13* (a class D gene), are important for floral meristem determinacy [34]. Furthermore, it is recently reported that the two class C genes interacts with *OsMADS16* (a class B gene) in suppressing indeterminate growth within the floral meristem [35]. Interestingly, carpel identity in rice is determined by a *YABBY* gene named *DROOPING LEAF* (*DL*) [31,36].

### 2.4. Rice Class D Genes

Analyses of expression and of protein-protein interactions suggested that the rice class D gene *OsMADS13* is involved in specifying ovule identity [37,38]. Recent mutation and los-of-function studies of *OsMADS13* revealed that it controls ovule specification [39,40]. Mutation of *OsMADS21*, a paralog of *OsMADS13*, does not result in any additive ovule defect, indicating that *OsMADS21* has lost its ability to determine ovule identity [34,39].

### 2.5. Rice Class E Genes

The class E genes of rice belong to two clades, the *SEP*-clade and the *LOFSEP*-clade [41]. In the *SEP*-clade, *OsMADS7*/*OsMADS45* and *OsMADS8*/*OsMADS24* show high sequence similarity to *Arabidopsis SEP* genes [41]. Simultaneous suppression of *OsMADS7* and *OsMADS8* causes severe meristic and homeotic effects in the inner three floral whorls; in particular, lodicules are transformed into lemma/palea-like structures [42].

The *LOFSEP*-clade contains *OsMADS1*/*LEAFY HULL STERILE 1* (*LHS1*), *OsMADS5*/*OSM5*, and *OsMADS34*/*PANICLE PHYTOMER 2* (*PAP2*) [43]. Mutation of *OsMADS1* in rice produces the *leafy hull sterile 1* (*lhs1*) phenotype that has a leaf-like lemma and palea, and lemma/palea-like lodicules [44]. Furthermore, knockdown of *OsMADS1* induces the transformation of the lemma into a glume-like structure [45]. These results indicate that *OsMADS1* functions in lemma and palea differentiation. In contrast with *OsMADS1*, mutation of *OsMADS34* developed altered inflorescence morphology with altered numbers of primary and secondary branches [46]. These indicate that *OsMADS34* and *OsMADS1* play important functions in specifying the inflorescence and spikelet. Recently, it was reported that *OsMADS34* acts in the shoot apical meristem together with the three *AP1*/*FUL*-like genes, *OsMADS14*, *OsMADS15* and *OsMADS18*, to specify the identity of the inflorescence meristem [47]. Simultaneous silencing of *LHS1*, *OsMADS5*, *OsMADS7*, and *OsMADS8* is sufficient to transform all floral organs, except the lemma, into leaf-like structures indicating that the four genes act in concert to provide a class E function in rice [42].

The rice *AGL6*-clade gene, *OsMADS6*/*MOSAIC FLORAL ORGANS 1* (*MFO1*), regulates floral organ identity, suggesting that it also has an E class function [48,49]. Another *AGL6*-clade gene, *OsMADS17*, has a minor but redundant function with that of *MFO1*. Recently, mutant analyses indicated that *OsMADS6* plays synergistic roles in floral organ specification with class B, C, D genes and with *DL* [50]. Furthermore, a null allele of *OsMADS6* exhibited transformation of floral organs except for lemma into lemma-like organs [51], indicating that *OsMADS6* acts as a critical regulator for floral organ formation.

## 3. Pistillody, Homeotic Transformation of Stamens into Pistil-like Structures, in the Alloplasmic Wheat Line

To investigate the effects of cytoplasm from wild relatives of common wheat (*Triticum aestivum*) on floral development, cytoplasmic substitution (alloplasmic) lines have been produced by recurrent backcrossing [52,53]. In an alloplasmic line in which *Aegilops crassa* cytoplasm has been introduced into the wheat cultivar (cv.) Norin 26 (N26), male sterility occurs under long-day conditions (>15 h light period) due to pistillody, the homeotic transformation of stamens into pistil-like structures (Figure 1) [54]. This phenomenon was named photoperiod-sensitive cytoplasmic male sterility (PCMS) and has been extensively investigated to assess its value to hybrid wheat breeding [55]. In contrast to N26, the wheat cv. Chinese Spring (CS) does not show pistillody when *Ae. crassa* cytoplasm is introduced; the absence of an effect is due to a single dominant gene (designated *Rfd1*) located on the long arm of chromosome 7B [56]. The role of *Rfd1* has been investigated by a loss-of-function analysis in an alloplasmic line of CS with ditelosomy of chromosome 7BS, *i.e.*, lacking the long arm of chromosome 7B, and with *Ae. crassa* cytoplasm {(cr)-CSdt7BS}. These plants showed pistillody indicating that the absence of *Rfd1* induces the phenotype irrespective of photoperiod. By contrast, CS plants with ditelosomy of 7BS but with a normal cytoplasm (CSdt7BS) form normal stamens [57]. These results indicate that pistillody is induced by factor(s) in the *Ae. crassa* cytoplasm, presumably from mitochondrial gene(s), and that the nuclear *Rfd1* gene prevents the deleterious effects of the cytoplasm. PCMS in the alloplasmic lines of N26 suggests the presence of an *Rf* gene that functions under short-day conditions. One candidate for the *Ae. crassa* cytoplasmic factor causing pistillody in alloplasmic wheat is the mitochondrial gene *orf260* [58]. It is also possible that retrograde (mitochondrion to nucleus) signaling via a protein kinase and calmodulin-binding protein may be involved in pistillody induction [59,60]. In the alloplasmic line, an ectopic ovule differentiates in the pistil-like stamens [54,57]. The pistillody line (cr)-CSdt7BS and the corresponding normal line CSdt7BS are useful for investigating the molecular mechanism of the homeotic change of stamens into pistil-like structures with an ectopic ovule induced by a cytoplasmic factor, and for identification of class BCD MADS-box genes. The functions of wheat class BCD MADS-box genes in detail would be examined by the transgenic studies.

## 4. Pistillody Reveals the Function of Class BCD MADS-Box Genes in Wheat

### 4.1. Wheat Class B Genes

In the ABCDE model, the loss-of-function in class B MADS-box genes (*AP3* and *PI* in *Arabidopsis*) results in pistillody, the homeotic transformation of stamens into carpel/pistil-like structures. The highly homologous wheat *AP3*-type genes, *TaMADS#51* and *TaMADS#82*, were the first Class B MADS-box genes to be identified [61]. Bread wheat is a hexaploid with the genomic constitution AABBDD in which each genome originated from a different ancestral species. The A genome is believed to derive from *T. urartu*, the B genome from *Aegilops speltoides* or another species in the Sitopsis section, and the D genome from *Ae. tauschii* [62]. Allopolyploidization leads to the generation of duplicated homoeologous genes (homoeologs) and, consequently, the hexaploid wheat genome contains triplicated homoeologs derived from the three ancestral diploid species. *TaMADS#51* and *TaMADS#82* are wheat homoeologs of the *AP3* ortholog (wheat *APETALA3*: *WAP3*) and are located on chromosomes 7B and 7D, respectively. A northern blot analysis showed that expression of *WAP3* is restricted to young spikes at the floral organ developing stage, suggesting that *WAP3* functions in floral organ formation [61]. The level of expression of *WAP3* is reduced in the pistillody line compared to the normal line [57]. *WAP3* has also been called *TaAP3* [63].

Two *PI*-type genes have been identified in wheat, namely *WPI1* (wheat *PISTILLATA1*) and *WPI2* [64]. A phylogenetic analysis using the deduced amino acid sequences indicated that *WPI1* and *WPI2* are orthologs of the rice *PI*-type genes *OsMADS4* and *OsMADS2*, respectively. *WPI1* and *WPI2* have also been called *TaPI-1* and *TaPI-2/TaAGL26*, respectively [63,65].

An *in situ* expression analysis showed that *WPI* and *WAP3* are expressed in the primordia of the stamen and lodicule in the normal wheat line; however, no transcripts were detectable in the pistil-like stamens of the pistillody line [64]. This finding indicates that pistillody results from a deficit of *WPI* and *WAP3* expression in whorl 3, suggesting that these genes have a class B function. 

### 4.2. Wheat Class C Genes

The *AG* orthologs of wheat, *WAG1* (wheat *AGAMOUS1*) and *WAG2*, were identified as class C genes [66,67]. The level of transcription of *WAG* genes is low at the early stages of initiation of floral organ primordia and at its highest at the booting to heading stages. An *in situ* expression analysis indicated that *WAG* genes are associated with pistil and pistilloid stamen formation in the alloplasmic line [68]. A phylogenetic analysis using the deduced amino acid sequences showed that *WAG1* and *WAG2* are orthologs of the rice *AG*-type genes, *OsMADS58* and *OsMADS3*, respectively [67,69]. *WAG1* and *WAG2* are also called *TaAG-1* and *TaAG-2/TaAGL39*, respectively [63,65].

### 4.3. Wheat Class D Genes

Two studies in wheat have identified five genes, *TaAGL2*, *TaAGL9*, *TaAGL31*, *TaAG-3A* and *TaAG-3B* as candidate orthologs of the rice class D gene, *OsMADS13* [63,65]. Subsequent sequence analyses showed that *TaAG-3A* is identical with *TaAGL9*, and *TaAG-3B* is identical with *TaAGL2*. Furthermore, *TaAGL2*, *TaAGL9* and *TaAGL31* show very high sequence similarity suggesting that may be homoeologous. These wheat orthologs of *Arabidopsis* *STK* have been renamed as *WSTK* (wheat *SEEDSTICK*) [68].

In alloplasmic wheat, ectopic expression of the class D gene *WSTK* occurs in the adaxial region of pistil-like stamens and ectopic ovule primordia are initiated in these regions [68]; this suggests that *WSTK* expression is involved in ectopic ovule formation in pistil-like stamens. In *Arabidopsis*, *STK* functions in ovule development by an interaction with the class C-lineage MADS-box genes, *AG*, *SHP1* and *SHP2*, which is mediated by the class E gene *SEP3* [8]. In the pistil-like stamens of alloplasmic wheat, ectopic expression of the class C MADS-box genes, *WAG1* and *WAG2*, and the class D gene *WSTK* is induced [68]. Furthermore, WSTK protein forms a complex with the class E protein, WSEP, but not with the class C proteins WAG1 and WAG2 [68]. These facts suggest that *WSTK* has a class D function in wheat, similar to *STK* in *Arabidopsis*.

### 4.4. Wheat DLOOPING LEAF Gene, TaDL

In rice, carpel (pistil) specification is regulated by the *DROOPING LEAF* (*DL*) gene that encodes a YABBY transcription factor [36]. *TaDL*, a *DL* ortholog in wheat, was identified by homology screening [70]. *In situ* expression analysis in the pistillody line showed that *TaDL* is expressed in the primordia of pistil-like stamens as well as in the pistil. This suggests that *TaDL* functions in specification of the pistil. Together with the observation that class B genes are not detected in the primordia of pistil-like stamens [64], these facts suggest mutual repression between *TaDL* and class B genes.

## 5. Other Homeotic Genes in Wheat

### 5.1. Wheat Class E Genes

With regard to *SEP*-like genes, two MADS-box genes, *WSEP* (*wheat SEPALLATA*) and *WLHS1* (*wheat LEAFY HULL STERILE 1*) have been identified in wheat [71]. Phylogenetic analysis showed that *WSEP* clusters in the same group as *OsMADS24* and *OsMADS45*. *In situ* hybridization experiments showed that *WSEP* is expressed in the inner three whorls (lodicules, stamens and pistils) at the floral organ differentiation stage. Interestingly, after floral organ identities have been determined, strong expression of *WSEP* is observed in the palea, suggesting that *WSEP* genes are not only involved in floral organ differentiation but also in their subsequent development. The palea-specific expression was also observed in rice *OsMADS6* (an *AGL6*-like gene), suggesting the unique role of class E gene in grasses [72]. Yeast two- and three-hybrid analyses indicated that WSEP forms a complex with wheat class B and C genes [71], in a similar fashion to *Arabidopsis SEP3* [73].

In addition to *WSEP*, *TaMADS1* has been identified and characterized as a wheat class E gene [74]. A phylogenetic study indicated that *WSEP* is an ortholog of rice *OsMADS45* and that *TaMADS1* corresponds to *OsMADS24*; this suggests that *SEP* orthologs have diverged into two groups in monocot species [71]. Transgenic *Arabidopsis* plants over-expressing *TaMADS1* show early flowering and terminal flower formation [74]. Although protein-protein interactions involving *TaMADS1* and wheat class B or C genes have not yet been examined, *TaMADS1* may have a similarity as *WSEP*, because over-expression of *WSEP* in *Arabidopsis* causes early flowering and terminal flower formation [71].

Based on phylogenetic studies, *WLHS1* is a wheat ortholog of *OsMADS1* [71], a member of *LOFSEP*-clade. Transcripts of *WLHS1* accumulate at high levels in the glume, lemma and palea, and at a low level in the pistil and stamen. It has been reported that *OsMADS1-like gene* expression in inflorescences varies among grasses such as *Sorghum bicolor*, *Chasmanthium latifolium*, *Avena sativa*, and *Pennisetum glaucum* [75]. The differences in the expression patterns of *OsMADS1*-like genes in wheat and other grass species may be associated with differences in the structures of their respective inflorescences.

In wheat, five genes, *TaMADS#12*, *TaAGL37*, *TaAGL6-1A*, *TaAGL6-1B* and *TaAGL-1C*, have been identified as candidate orthologs of *AGL6*-like genes [61,63,65]. *TaMADS#12* and *TaAGL6-1B* are identical, as are *TaAGL37* and *TaAGL6-1A*; this suggests that these genes are homoeologs. The function of wheat *AGL6*-like genes has yet to be ascertained.

### 5.2. Wheat Class A Genes

*Arabidopsis* has two class A genes, *AP1* and *AP2*. The *AP1* MADS-box gene functions in the specification of floral meristem identity and in the determination of sepal development. There are two other *AP1*-like genes, *FRUITFULL* (*FUL*) and *CAULIFLOWER* (*CAL*), which have redundancy of function in specification of floral meristem identity with *AP1* [76]. Sequence analysis of *AP1*-like genes in monocots suggests that they only have FUL-like proteins, in contrast to dicot species, which have AP1, FUL and CAL proteins [22].

The grass family genome has three paralogs of *AP1/FUL-*like genes, namely, *FUL1* (corresponding to *VERNALIZATION1* (*VRN1*) in wheat), *FUL2* and *FUL3*, which are all derived from the *FUL* lineage [22]. Wheat *FUL1*, *WFUL1/VRN1*, has no class A function but acts in phase transition from vegetative to reproductive growth [77,78,79,80]. A phylogenetic analysis using the deduced amino acid sequences showed that *WFUL1*, *WFUL2* and *WFUL3* are orthologs of the rice *AP1*-type genes, *OsMADS14*, *OsMADS15* and *OsMADS18*, respectively [81].

In young spikes, expression of *WFUL2* is greatly reduced in stamens and cannot be detected in pistils, whereas *WFUL1/VRN1* and *WFUL3* are expressed in all floral organs [81], suggesting that *WFUL2* has a different function in the outer floral organs (lemma and palea) compared to the inner floral organs (stamen and pistil). Yeast two- and three-hybrid analyses showed that WFUL2 interacts with class B and class E proteins [81]. In combination with the expression analyses, these observations suggest that *WFUL2* specifies the identity of the outer floral organs in the wheat floret. In rice, both FUL1 and FUL2 proteins (OsMADS14 and OsMADS15, respectively) interact with a class E protein (OsMADS1/LHS1) [82], suggesting that the diversification of function between FUL1 and FUL2 detected in wheat has not occurred in rice. Especially, it is notable that wheat *FUL1* (*WFUL1/VRN1*) has important role at leaves as well as at shoot apex in flowering [81]. Expression and protein-protein interaction studies suggested that *WFUL2* in wheat has a class A function in development of the outer floral organs (lemma and palea) in combination with class B and class E MADS-box genes [81].

The wheat *Q* gene has been identified as an *AP2*-like gene [83]. The *q* allele confers a ‘speltoid’ spike phenotype that is characterized by a loosely formed head structure with elongated rachis and non-free-threshing seed. A phylogenetic analysis found that *Q* is not orthologous to *Arabidopsis AP2*; rather, another *AP2*-like gene, *TaAP2*, is the *AP2* ortholog [84]. The barley *AP2* ortholog, *HvAP2/Cly1* is associated with lodicule development [85], suggesting that *TaAP2* in wheat functions in floral organ formation, especially in lodicule development. Together with the observations in rice *AP2*-like genes [26,27], these findings may imply that the *AP2*-like gens in grasses have common function in floral organ formation.

## 6. Wheat ABCDE Model, Complicated Homoeologous Gene Interaction

**Figure 3:**
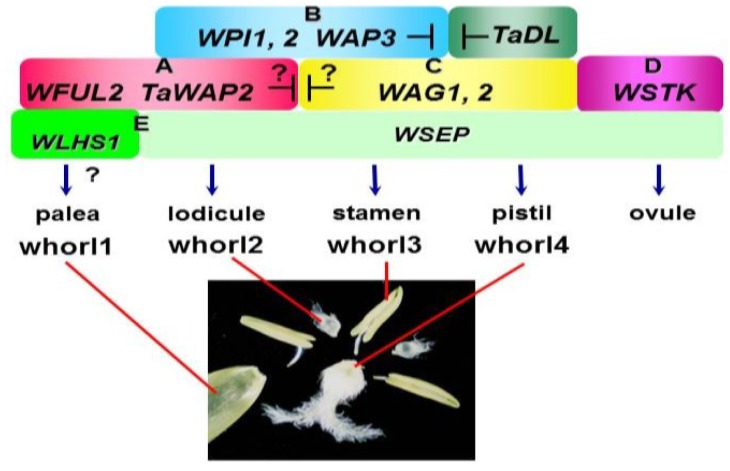
The ABCDE model of floral organ formation in wheat. In contrast to the *Arabidopsis* ABCDE model, the wheat ABCDE model involves duplicated genes for class B (*PI*-like) and class C (*AG*-like) functions. Furthermore, class E genes are divided into two groups, *WSEP* and *WLHS1*, with sub-functionalization. A *YABBY* gene *TaDL* specifies the pistil (carpel) identity. The pistillody line has been valuable for constructing the ABCDE model in wheat. The wheat ABCDE model is similar to that of rice except for the class A genes. The current wheat ABCDE model indicates that class B and TaDL proteins show mutual suppression, which was suggested from analysis of a pistillody line. The mutual suppression between class A and C genes is also postulated here. The wheat ABCDE model probably functions through complex homoeologous gene interactions.

The wheat ABCDE model for floral organ formation is illustrated in Figure 3. The relationships of homeotic genes among *Arabidopsis*, rice and wheat are shown in Table 1. As mentioned earlier, wheat is an allohexaploid species with the genome constitution AABBDD. Consequently, the hexaploid wheat genome contains triplicated homoeologs derived from the three ancestral A, B and D genomes. There are three possible evolutionary fates for homoeologs in polyploids: functional diversification, gene silencing, and retention of original or similar function [86]. Functional diversification of homoeologs is one of the important factors in the evolutionary success of polyploid species [87]. 

With regard to class E genes, analyses of gene structure, expression patterns and protein functions showed no evolutionary changes to the *WSEP* homoeologs. In contrast, the three *WLHS1* homoeologs show genetic and epigenetic alterations [71]. The A genome *WLHS1* homoeolog (*WLHS1-A*) has a large deletion in the region of the K domain sequence. Data from a yeast two-hybrid analysis and a transgenic experiment indicated that the WLHS1-A protein does not have a function in floral development. *WLHS1-B* and *WLHS1-D*, located in the B and D genomes, respectively, have the complete MADS-box gene structure; however, *WLHS1-B* is effectively silenced by epigenetic regulation. Consequently, of the three homoeologs, only *WLHS1-D* functions in hexaploid wheat.

The example of the *WLHS1* genes indicates the possibility that homoeologs of each homeotic gene may be differentially regulated in wheat spike formation. Floral homeotic MADS domain proteins interact in floral tissue as proposed in the “floral quartet” model, in which a tetramer of MADS domain proteins functions in specification of floral organ identity [2,73]. The complex homoeologous gene interactions are probably associated with morphological, physiological and ecological diversification among different ploidy levels. Polyploid wheat must be a good model for investigating this point [88].

**Table 1:** The relationships of homeotic genes among *Arabidopsis*, rice and wheat.

Class	Clade	Arabidopsis	Rice	Wheat
class A		*AP1*	*OsMADS14/RAP1B*	*WFUL1/VRN1*
			*OsMADS15/RAP1A*	*WFUL2*
			*OsMADS18*	*WFUL3*
		*AP2*	*SNB*	*TaAP2*
			*OsIDS1*	*Q*
			*MFS1*	
class B		*AP3*	*OsMADS16/SPW1*	WAP3/TaAP3 *
		*PI*	*OsMADS2*	*WPI2/TaPI-2/TaAGL26*
			*OsMADS4*	*WPI1/TaPI-1*
class C		*AG*	*OsMADS3*	*WAG2/TaAG-2/TaAGL39*
			*OsMADS58*	*WAG1/TaAG-1*
class D		*STK*	*OsMADS13*	WSTK **
		*SHP1, 2*		
class E	*SEP*	*SEP1, 2, 3, 4*	*OsMADS7/OsMADS45*	*WSEP*
			*OsMADS8/OsMADS24*	*TaMADS1*
	*LOFSEP*		*OsMADS1/LHS1*	*WLHS1*
			*OsMADS5/OsM5*	
			*OsMADS34/PAP2*	
	*AGL6*	(*AGL6*)	*OsMADS6/MFO1*	TaAGL6 ***
			*OsMADS17*	
other		(*CRC*)	*DL*	*TaDL*

* *TaMADS#51* and *TaMADS#82* are two of three homoeologs of *WAP3*; ** *TaAGL2/TaAG-3B*, *TaAGL9/TaAG-3A* and *TaAGL31* are homoeologs of *WSTK*; *** *TaAGL6-1A/TaAGL37*, *TaAGL6-1B/TaMADS#12* and *TaAGL6-1C* are homoeologs of *TaAGL6*.

## Abbreviations

*AG*: *AGAMOUS*
*AGL*: *AGAMOUS LIKE*
*AP*: *APETALA*
CS: Chinese Spring
*DL*: *DROOPING LEAF*
*FUL*: *FRUITFULL*
*LHS*: *LEAFY HULL STERILE*
*MFO*: *MOSAIC FLORAL ORGAN*
*MFS*: *MULTI-FLORET SPIKELET*
N26: Norin 26
*OsIDS*: *Os INDETERMINATE SPIKELET*
*PAP*: *PANICLE PHYTOMER*
*PI*: *PISTILLATA*
*SHP*: *SHATTERPROOF*
*SEP*: *SEPALLATA*
*SNB*: *SUPERNUMERARY BRACT*
*SPW*: *SUPERWOMAN*
*STK*: *SEEDSTICK*
*VRN*: *VERNARIZATION*
